# NeuronBridge: an intuitive web application for neuronal morphology search across large data sets

**DOI:** 10.1186/s12859-024-05732-7

**Published:** 2024-03-15

**Authors:** Jody Clements, Cristian Goina, Philip M. Hubbard, Takashi Kawase, Donald J. Olbris, Hideo Otsuna, Robert Svirskas, Konrad Rokicki

**Affiliations:** Janelia Research Campus, Howard Hughes Medical Institute, Ashburn, USA

**Keywords:** Drosophila, Connectomics, Cloud computing, Serverless, Web service, Database, Open data API

## Abstract

**Background:**

Neuroscience research in *Drosophila* is benefiting from large-scale connectomics efforts using electron microscopy (EM) to reveal all the neurons in a brain and their connections. To exploit this knowledge base, researchers relate a connectome’s structure to neuronal function, often by studying individual neuron cell types. Vast libraries of fly driver lines expressing fluorescent reporter genes in sets of neurons have been created and imaged using confocal light microscopy (LM), enabling the targeting of neurons for experimentation. However, creating a fly line for driving gene expression within a single neuron found in an EM connectome remains a challenge, as it typically requires identifying a pair of driver lines where only the neuron of interest is expressed in both. This task and other emerging scientific workflows require finding similar neurons across large data sets imaged using different modalities.

**Results:**

Here, we present NeuronBridge, a web application for easily and rapidly finding putative morphological matches between large data sets of neurons imaged using different modalities. We describe the functionality and construction of the NeuronBridge service, including its user-friendly graphical user interface (GUI), extensible data model, serverless cloud architecture, and massively parallel image search engine.

**Conclusions:**

NeuronBridge fills a critical gap in the *Drosophila* research workflow and is used by hundreds of neuroscience researchers around the world. We offer our software code, open APIs, and processed data sets for integration and reuse, and provide the application as a service at http://neuronbridge.janelia.org.

## Background

Advances in serial-section electron microscopy technologies have enabled the generation of comprehensive nervous systems maps, known as connectomes, of the *Drosophila melanogaster* central nervous system [1–7]. These connectomes are derived from segmented EM images which are reconstructed to describe the precise structure of the neurons in a brain, as well as the connections (i.e., synapses) between them. Neuroscientists use connectomic information to relate neuronal connectivity structure with how neurons function *in vivo*. Here we focus on two large connectomes produced by Janelia’s FlyEM project team: the FlyEM Hemibrain data set [4], which covers more than half of a female adult fly brain, and the FlyEM Male Adult Nerve Cord (MANC) data set [6]. Together these data sets cover a large portion of the fly nervous system.

Importantly, neuron morphology is highly conserved across different specimens in *Drosophila* [8], enabling the study of selected neuronal circuits across individuals. Researchers target one or more carefully selected neurons for visualization, neuronal activity measurement, genetic modification, ablation, or stimulation, and combine these tools with animal behavior studies. Janelia’s FlyLight project team produced a large driver line library known as the Generation 1 (Gen1) GAL4 Collection [9], which provides the starting point for targeting specific neurons. Each driver line expresses the GAL4 protein under control of a genomic enhancer fragment. When crossed to another line with a UAS (Upstream Activating Sequence) reporter gene, GAL4 binds to UAS and activates reporter expression in defined neurons [10].

**Fig. 1:**
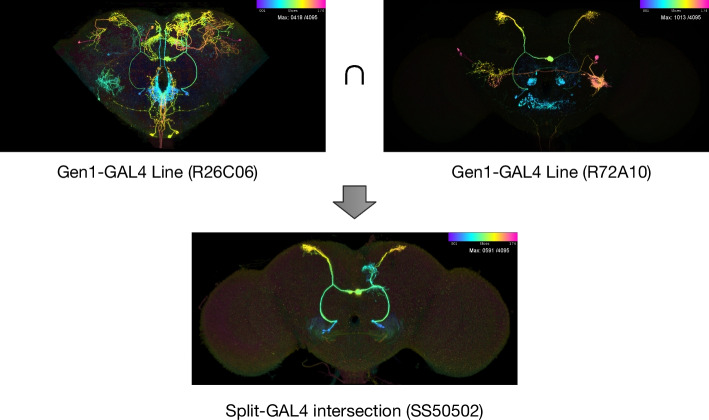
Genetic tools for *Drosophila*. Two Gen1 driver lines with broad expression are crossed using Split-GAL4 to create a new driver line with the intersection of the parent lines’ expression pattern. Images are color depth maximum intensity projections (CDM), where the blue color indicates the anterior of the brain and red the posterior. Upper section shows one of three MCFO channels as a CDM. Lower section shows the full Split-GAL4 expression pattern from driver line SS50502 [24]. Original 3D images are available at https://gen1mcfo.janelia.org and https://splitgal4.janelia.org

To provide greater specificity and target individual neurons, the two-component Split-GAL4 method [11, 12] is used to produce an expression pattern reflecting only the common elements in the patterns of two driver lines; i.e., an intersection between two expression patterns is performed. Ideally, this results in a new driver line that targets a single neuron or cell type (Fig. 1). Subsequently, the GAL4/UAS system [10] allows further genetic modifications of the targeted neurons (fluorescence, ablation, etc.).

Creating an effective Split-GAL4 line requires the careful identification of two parent Gen1 GAL4 lines which have expression in the same neuron of interest with no other expression in common. Finding such candidate lines in a collection of thousands of images is a labor-intensive process and benefits from a computationally assisted workflow [13, 14]. The FlyLight Gen1 GAL4 driver collection has been characterized recently [15] with confocal imaging of neurons isolated by stochastic multi-color labeling (MCFO; [16]). This allows for the visualization and identification of individual neurons and provides a basis for a computational workflow for Split-GAL4 creation [15].

Increasing numbers of neuron and cell-type specific Split-GAL4 driver lines are being characterized [17–36]. FlyLight recently released a large collection of cell-type-specific Split-GAL4 driver lines [37]. As the number of published Split-GAL4 driver lines increases, a second workflow will become important: searching these lines to find ready-made drivers for neurons identified in a connectome.

In other common workflows, researchers traversing circuits in a connectome want to verify cell type identity by referencing known driver lines [26, 33, 38], or by finding additional neurons of the same cell type with a similar morphology to a known neuron of interest [28]. In yet another workflow, a researcher starts with a neuron imaged in LM and looks for that neuron in a connectome [36].

All of the above workflows require identifying similar neuron morphology across large data sets of EM and LM images. Registration of neurons to a common reference alignment space enables spatial matching across biological samples and modalities [39]. Two current solutions to the computational problem of matching neurons across registered imaging modalities are Color Depth MIP Search (abbreviated as CDM Search; [13]) and PatchPerPixMatch (PPPM; [14]). CDM Search represents location in the projection dimension as color in a maximum intensity projection (MIP) and thus enables efficient 3D structure comparison through simple 2D similarity between overlapping pixels. PPPM leverages deep-learning segmentation (PPP; [40]) of the LM images and an algorithm based on NBLAST [41] to find the best matching neuron fragments (from LM) that fit a reconstructed neuron skeleton (from EM) in 3D space. A comparison of CDM Search and PPPM is available elsewhere [15].

However, the CDM and PPPM algorithms are not readily accessible to experimentalists. CDM Search was initially implemented as a Fiji plugin [42] for executing searches locally. Therefore, it requires the user to download large data sets and wait for each search to execute using local compute resources. PPPM provides precomputed matches for a subset of FlyLight Gen1 MCFO images against the FlyEM Hemibrain and MANC, but the algorithm is expensive to run ($\sim$3 h per LM image on a single GPU) and is not easily usable for custom searching with user data. To make these algorithms more accessible, we built a web application that experimentalists can use to rapidly identify and view similar neurons in published EM and LM data sets, or to perform searches on their own data. The only comparable software that we know of is the NBLAST functionality in Virtual Fly Brain ([43]), which allows users to find similar neurons across the FlyCircuit data set. However, it is currently limited to data sets where the neurons have been traced as skeletons, so it does not address the important use case of searching large amounts of LM data containing neurons which are difficult to trace automatically.

## Implementation

To address the problem of finding similar neurons across large multi-modal data sets, we developed NeuronBridge, an easy-to-use web application. It provides instant access to neuron morphology matches for published EM and LM data sets, as well as rapid custom searching against those data sets. Our initial implementation was based on the CDM Search algorithm and PPPM results were added later. Implementing CDM Search in a publicly available web application with interactive response times presented several major challenges: (a) the CDM Search tools were only available within Fiji [42] and lacked programmatic APIs for reuse; (b) when centralized and scaled to hundreds of users, CDM searching becomes expensive to run *de novo* for each query; (c) CDM Search relies on fast local disk access and multithreading, which are expensive resources to leave idle at the scale needed to support unpredictable usage access patterns; and (d) users seeking to search using their own data first need to align it to the standard template and generate aligned CDM images for their neurons.

We first extracted the CDM Search algorithm from its Fiji plugin, refactored it into a Java library (Software Availability), and made it available for programmatic reuse. To enable large-scale computation on a high performance computing (HPC) cluster, we used the CDM Search library to create an Apache Spark application for running the CDM Search algorithm in a distributed manner.

**Fig. 2:**
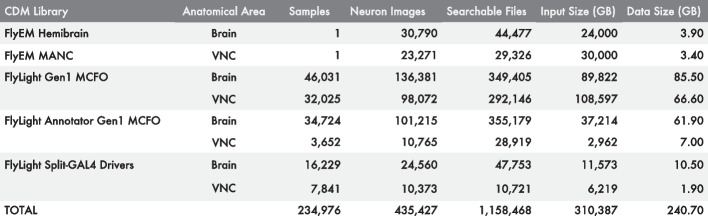
Data available in NeuronBridge. CDM libraries are loaded into NeuronBridge with precomputed results and are available for custom searching. *CDM Library* indicates the CDM library being described. *Anatomical Area* shows the region of the fly central nervous system covered by the library. *Samples* shows the number of biological samples which form the original data set. *Neuron Images* indicates the total number of individual images containing neurons derived from these samples. *Searchable Files* indicates the number of CDM images in the library that can be searched. *Input Size (GB)* indicates the total uncompressed size of the original data set. *Data Size (GB)* indicates the total size of the CDM images used for searching

Second, we generated collections of CDM images, henceforth referred to as “CDM libraries”, from Janelia’s published EM and LM data sets including the FlyEM Hemibrain [4], FlyEM MANC [6], FlyLight Gen1 MCFO and Annotator Gen1 MCFO [15], and the Split-GAL4 driver line collection (Fig. 2). To populate the CDM libraries, we generated aligned CDM images (Image Alignment) from FlyEM’s published EM neuron skeletons and from FlyLight’s published 3D image volumes. We then used our Apache Spark CDM Search implementation to precompute neuron matches between EM and LM libraries (Neuron Morphology Matching). Each EM CDM was compared with each LM CDM, a total of over 33 billion image-to-image comparisons for FlyEM Hemibrain, and 10 billion comparisons for the FlyEM MANC. Matches were recorded in both directions, to allow bi-directional searching. This step obviated the need to run *de novo* searches for matches between published data sets, mitigating the ongoing costs of running the shared service, and allowing us to make the service freely available to the community.

Third, we developed a data model (Data Model) for representing matches that we define as putatively similar neuronal morphologies identified with respect to two images that are registered to the same template. Our data model allows matches from CDM Search, PPPM, and potentially any future algorithms to be represented with the same entity classes, as well as allowing for an extensible set of imaging modalities, making it easier to add new data in the future while maintaining a consistent user experience.

Then we imported the published PPPM results [14] for the FlyEM Hemibrain, FlyEM MANC, and FlyLight Gen1 MCFO data sets into our data model alongside the CDM Search results. The PPPM results feature additional images for visualizing matches, including overlays of EM skeletons on top of masked LM signals, which we were able to import into our data model as new file types.

Next, we leveraged the Amazon Web Services (AWS) Open Data program to make all of the images and match metadata available on AWS S3 as a robust public data API (Open Data API). AWS S3 is a serverless object storage service with high reliability, low latency, access control, and tight integration with other AWS services. Using a serverless solution allowed us to keep costs low compared to a traditional relational database deployment. We also developed a simple Python module (Software Availability) on top of our API, to enable ad hoc data analysis through the use of Python libraries, and to serve as a client reference implementation.

Next, we created an online custom search service for user-uploaded data (Software Architecture) that (1) registers uploaded confocal images to a reference brain using the Computational Morphometry Toolkit (CMTK, [44]) running on AWS Batch, (2) generates a CDM image for each channel of the uploaded stack, (3) allows the user to select their neurons of interest with an interactive browser-based image masking tool, and (4) runs a massively parallel CDM Search to find neuron matches in available CDM libraries. It’s also possible for the user to directly upload their own aligned CDM image to use as the search target. Importantly, the custom search service is entirely serverless, which minimizes costs by not idling compute resources in the cloud. To make this possible we developed a burst-parallel compute engine (Software Architecture) to achieve on-demand, 3000-fold parallelism using the AWS Lambda service, which allows a search of the EM CDM libraries to complete in seconds and a search of the LM CDM libraries to complete in under two minutes.

**Fig. 3:**
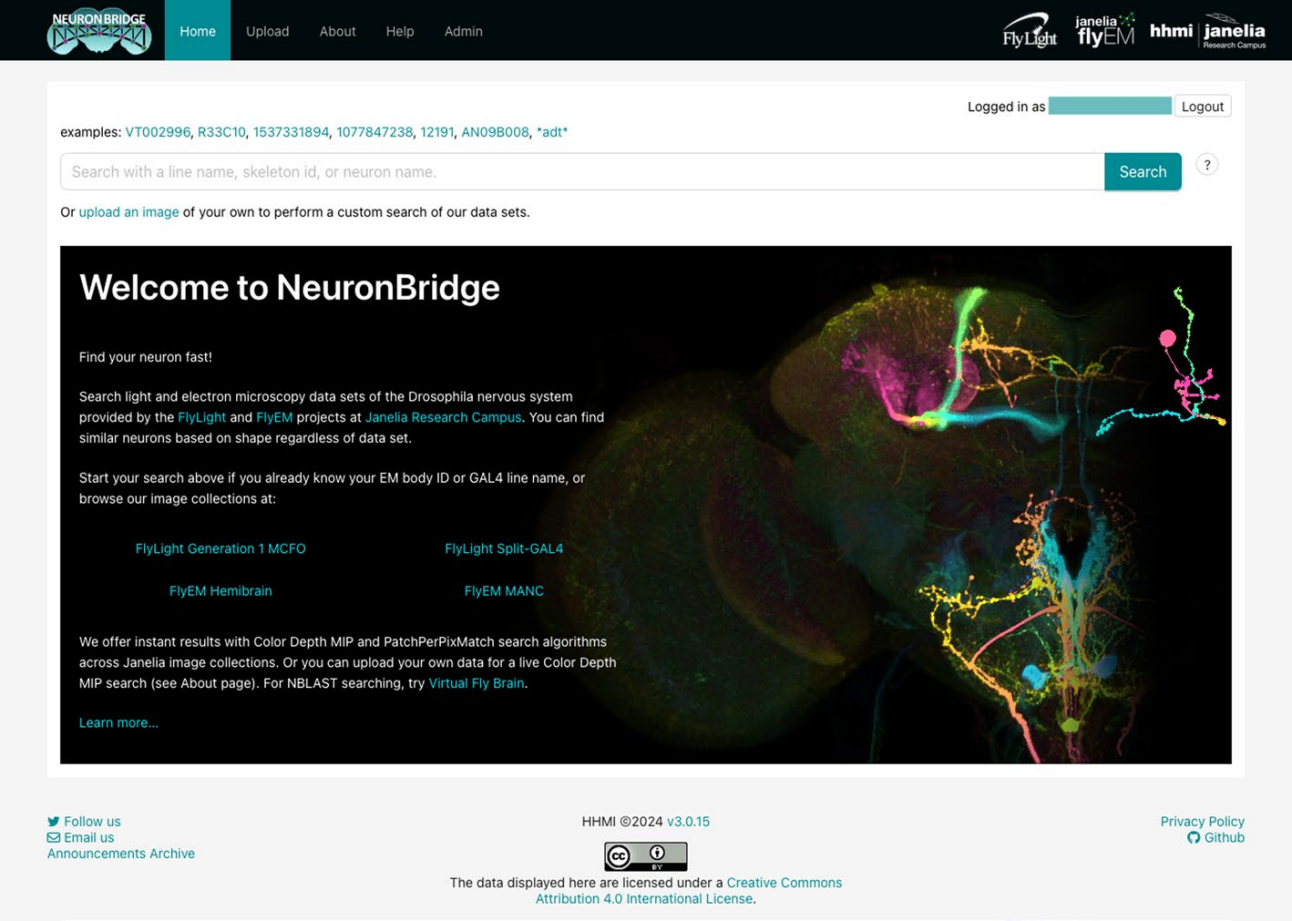
NeuronBridge home page. The search box enables quick access to EM and LM CDM images using common identifiers such as neuron identifiers and driver line names

Finally, we built a user-friendly, single-page application (SPA) using the React framework (User Experience), enabling end-users to browse precomputed matches and run searches with their own data. The graphical user interface (GUI) of the application (Fig. 3) was designed to be simple and intuitive, focusing solely on the task of neuron matching, providing contextual help information, and including usage examples where appropriate.

### Image alignment

**Fig. 4:**
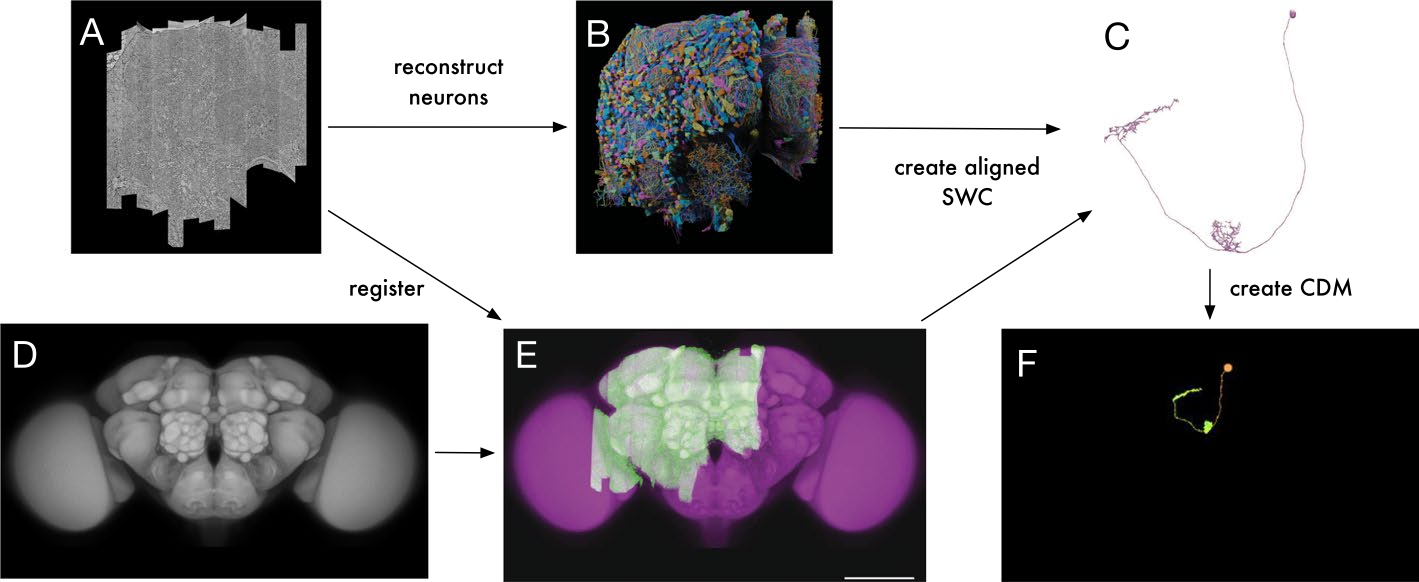
Pipeline for EM CDM generation. **A** FlyEM Hemibrain stitched data set; screenshot from Neuroglancer. **B** FlyEM Hemibrain reconstruction by Janelia FlyEM and Google. **C** Example neuron reconstruction (ID 1537331894) from the FlyEM Hemibrain; screenshot from neuPrint. **D** JRC2018 Unisex brain template. **E** Registration of FlyEM Hemibrain to the JRC2018 Unisex template. **F** CDM of the neuron shown in **C**

**Fig. 5:**
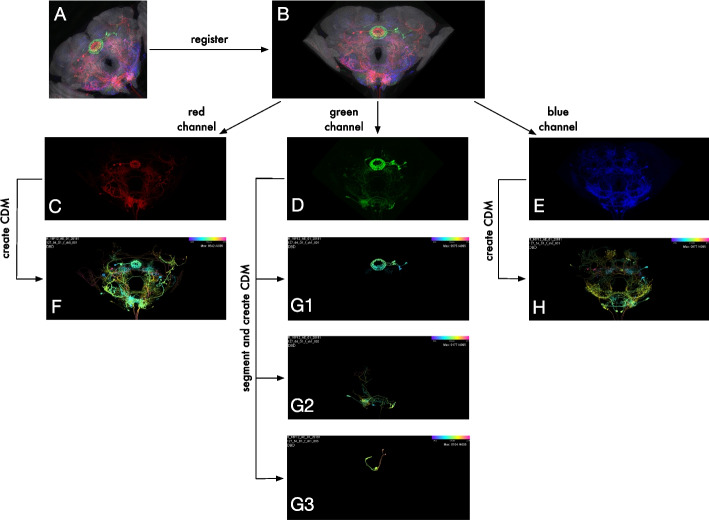
Pipeline for LM CDM generation. **A** MIP of an example LM MCFO image (slide code 20181127_64_D1) of a brain sample from the R16F12 driver line. **B** LM image registration to JRC2018 Unisex template. **C–E** Aligned MIPs of individual channels. **F–H** CDM images for each channel. **G1–G3** Segmentation of green channel using modified DSLT yields three voxel sets for the green channel. Each voxel set is converted into a separate CDM

For the FlyEM Hemibrain data set we relied on the published registration to the JRC2018 *Drosophila* female brain template [39] (Fig. 4A, D, E). For the FlyEM MANC, we used the published registration to the JRC2018 *Drosophila* male VNC template [6]. For the FlyLight data sets, we used CMTK [44] to register all LM images (Fig. 5A, B) to the sex-appropriate JRC2018 template [39]. In all cases we subsequently used a bridging transform to move the data into the alignment space of the JRC2018 Unisex template, where neuron matching could be performed across all samples, irrespective of sex.

For online custom searching, the alignment code is packaged as a Docker container and run using AWS Batch. For better performance, online searches skip the alignment to sex-specific templates and align directly to the JRC2018 Unisex template.

### Neuron morphology matching

The CDM Search algorithm [13] was designed for interactive use and required several modifications for reliable batch execution on large data sets [15]. These changes will be described fully in a future paper [45] but we provide a brief outline here.

First, we generated CDM libraries from all of the input data sets. To create the EM CDM libraries we started with neuron skeletons (Fig. 4C), which are approximations of neuronal morphology computed from the proofread segmentation of the EM image data [4]. Compared to the full neuron reconstructions, using the skeletons allowed us to control the diameter of the neurons for better CDM search results. We transformed the skeletons into the aligned space (Image Alignment) and generated a CDM image for each neuron (Fig. 4F).

While EM segmented neurons represent individual cells, biological GAL4 expressions typically exhibit bilateral symmetry [4]. To enhance matching between the FlyEM Hemibrain and LM data, we generated additional artificial CDM images. This was done by mirroring and combining the image with the original CDM for any neurons crossing the midline. These ‘flipped’ neurons expand the CDM image set, resulting in higher matching scores for bilateral neurons that express GAL4 on both sides of the brain in LM images. Nonetheless, due to the stochastic nature of MCFO, many MCFO samples express GAL4 on only one side of the brain. This type of asymmetric expression tends to yield higher matching scores with non-flipped, ‘original’ EM single neurons. To maximize the probability of matches in both cases, we compared the EM-LM matching scores between ‘flipped’ and ‘original’ versions for the same EM neuron and LM sample, selecting the higher score as the final matching result in NeuronBridge.

To create LM CDM libraries, we started with registered LM confocal images and generated multiple CDMs for each channel of every aligned LM image (Fig. 5C–H). LM images may contain tissue background signal which interferes with CDM Search matching. Also, denser fluorescence labeling in MCFO samples can produce occlusions of neurons. To address these issues, we performed a 3D neuronal segmentation of each channel using an algorithm based on Direction Selective Local Thresholding (DSLT, [46]), which resulted in a handful of voxel sets per channel (Fig. 5G). We eliminated smaller “junk” voxel sets based on manually chosen Fiji shape descriptor thresholds and generated CDM images of the remaining voxel subsets.

When running CDM Search, we used the images in the EM CDM library as search targets and compared each one with every image in the LM CDM libraries. We limited the number of LM matches to a maximum of 300 driver lines. The CDM matching was initially conducted only from the EM to the LM direction. Nevertheless, NeuronBridge enables users to conduct searches from LM to EM because we recorded the matching results for each LM sample across all EM neurons, based on the matching score.

To maximize the likelihood of the best matches rising to the top of the search results, we created a uni-directional shape-matching algorithm that is run after CDM Search to re-sort the results. The algorithm penalizes EM CDM pixels that are not within approximately $\pm 12$ microns of LM CDM pixels in the XY dimensions. It also penalizes more than 40 microns of unmatched EM CDM pixels in the Z dimension, within the $\pm 12$ micron XY region. It does not similarly penalize LM CDM pixels because LM samples typically express GAL4 in multiple neurons simultaneously, leading to expression patterns that do not align with single EM neurons. Ideally, if an EM neuron is contained within an LM sample, the LM image should cover all corresponding EM branches depicted in the LM data. However, if an incorrect EM neuron is matched with the LM sample, this will result in EM branches that are not represented in the LM sample, penalizing the match score. The final score for each match was computed as a ratio of positive matching (i.e., number of matched pixels) to negative matching (i.e., the penalties).

### User experience

NeuronBridge is an end-user web application designed for accessibility and ease-of-use by wet-lab biologists. Using any web browser, a user can log into the service’s website with their Google account credentials or create a separate NeuronBridge account. The user is presented with a simple search interface for looking up their neuron or GAL4 driver line of interest (Fig. 3). Searching for a neuron identifier returns CDM representations of EM neuron reconstructions, in the JRC2018 Unisex alignment space. Searching for a driver line returns CDM representations of that driver line. These initial search results are displayed in a tabular, paginated format, allowing the user to select an image of interest to begin neuron matching. Each result has buttons corresponding to the types of match algorithms that were used to compare it to the other modality, typically both CDM Search and PPPM. These buttons let the user view putative neuron matches with other images (LM matches for EM targets, EM matches for LM targets).

**Fig. 6:**
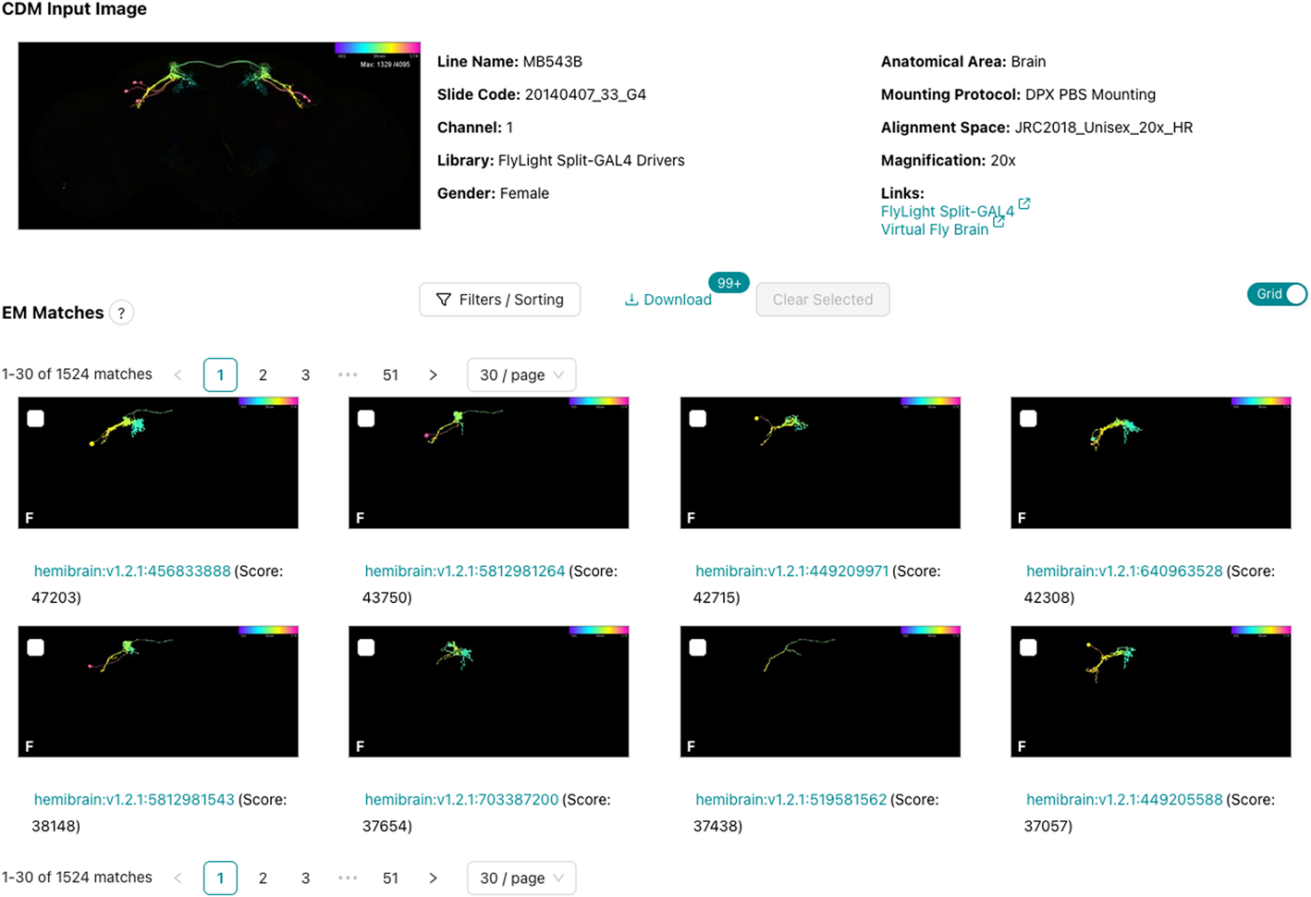
Browsing EM matches for an LM image. Paginated EM matches are displayed for the CDM input image shown at the top. The results can be filtered and sorted, or shown as a list. Checkboxes allow users to select promising matches for download and further analysis. Screenshot was truncated to only show the first two rows of results

After selecting a target image and an algorithm, precomputed neuron matches are immediately displayed as a list or a grid (Fig. 6) and paginated for rapid browsing. They can be filtered by various criteria, such as the CDM library that the image belongs to, or the driver line name. When searching for LM matches, all of the matches for a single driver line are grouped and sorted together by the score of the highest ranking representative of that line. These results can be filtered to display a maximum number of representatives per driver line. A setting of 1 is useful when the user is interested in finding candidate lines for creating a Split-GAL4 driver line.

**Fig. 7:**
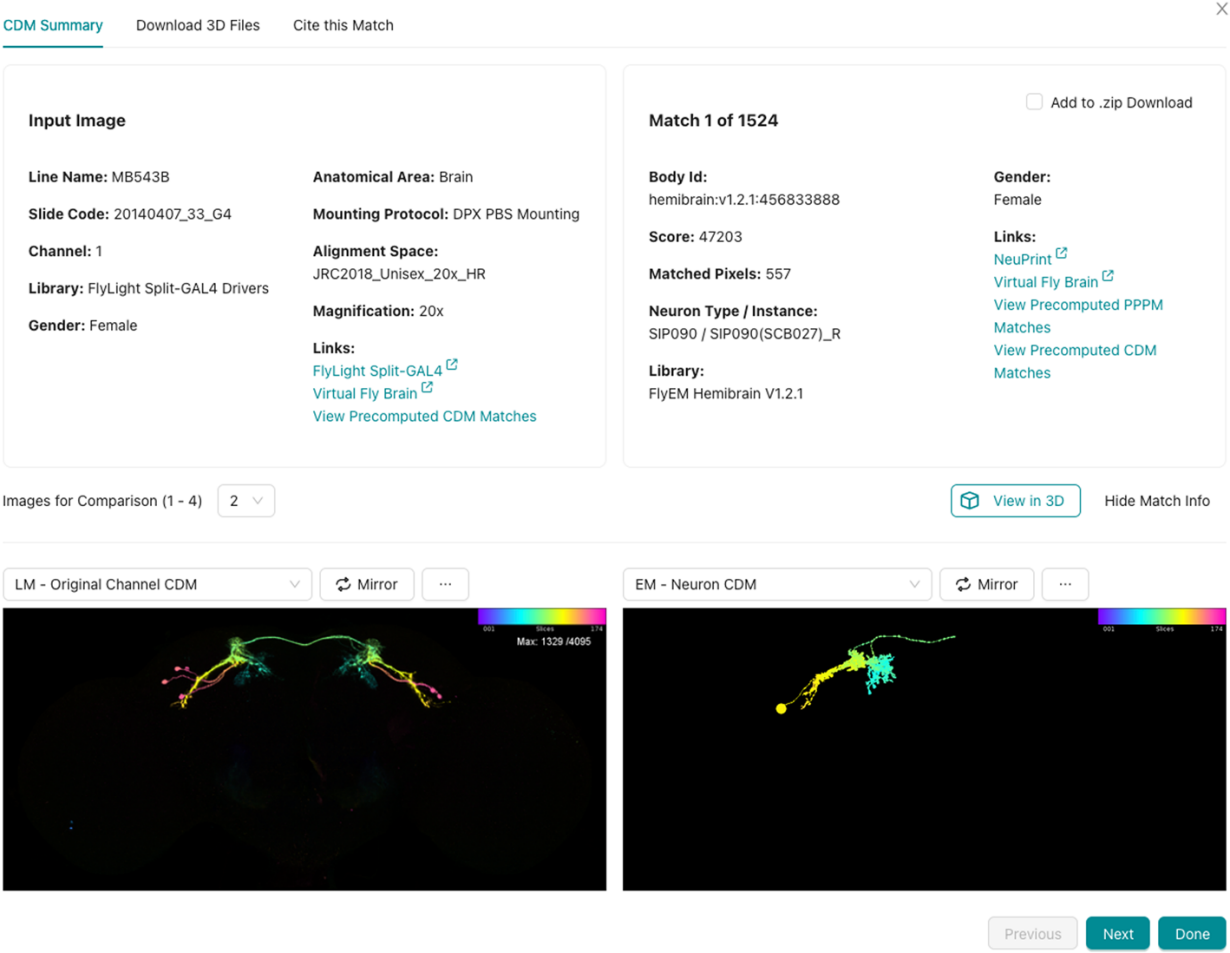
Details for a single EM/LM match. LM input image shown on the left and the EM match on the right. This view is customizable such that the user can choose how many images to display and select which representation to show in each screen location

Clicking one of the matches brings up a detail interface (Fig. 7) which allows the user to compare the match to the target using a mouse cursor synchronized across multiple images. Various accessory images can be displayed to better characterize the match and the driver line or connectome where it originated. Matches can be selected for download, from either the overview page or the detail page. The user can download all the metadata for the selected matches as a CSV file, and all of the matching images as a ZIP file.

**Fig. 8:**
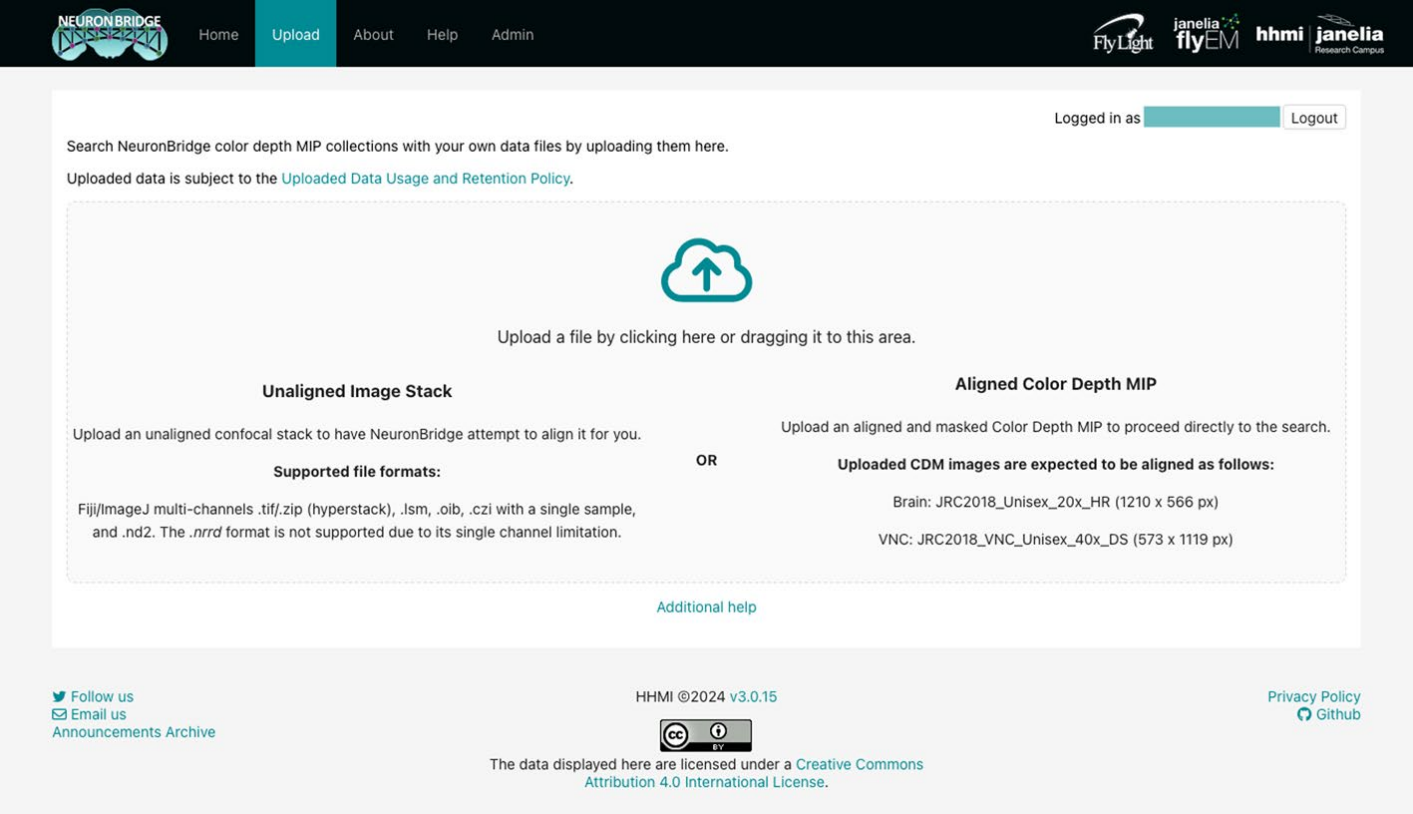
Uploading data for custom search. The upload GUI allows the user to drag and drop a file to begin the custom search workflow. Input data requirements are described in detail

**Fig. 9:**
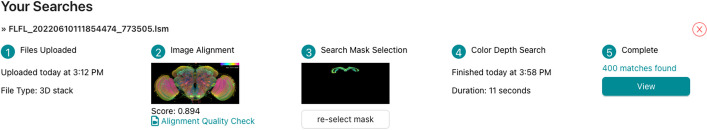
Custom search progress steps. The workflow can be branched by re-selecting the mask so that a different search can be run with the same input stack. This saves a considerable amount of time by reusing the alignment result

In addition to the precomputed match results between published images, NeuronBridge supports custom CDM Search against any of the EM and LM image libraries. Users begin a custom search by uploading their own image (Fig. 8), which can be an unaligned image stack in a variety of standard microscopy image formats (TIFF, ZIP, LSM, OIB, CZI, ND2), or an aligned CDM mask in the JRC2018 Unisex alignment space. NeuronBridge attempts to align uploaded images and generates a CDM for each channel. This process may take several minutes, and the user may wait for the alignment task to complete, or come back later to find their aligned CDMs. Next, the user is asked to choose a channel and create a search mask. The user may select the CDM libraries to search, and optionally specify other search parameters before invoking the search. A custom search typically takes less than a minute to process and progress is displayed in a step-wise workflow GUI (Fig. 9). Results are presented identically to how precomputed results are displayed.

The web GUI was developed using the React framework in order to make the GUI components reusable and more responsive than a traditional web application, by never fully reloading the page as one moves around the site. Instead, data is loaded via asynchronous HTTP requests, and the GUI is updated as data becomes available. By using AWS AppSync, we also extended this idea to asynchronous updates using Web Sockets, so that long-running back-end operations such as alignments can be monitored without polling. The single-page application (SPA) paradigm also allowed us to serve the website as static content, with caching via the AWS CloudFront Content Distribution Network (CDN) that speeds up initial loading.

### 3D visualization

**Fig. 10:**
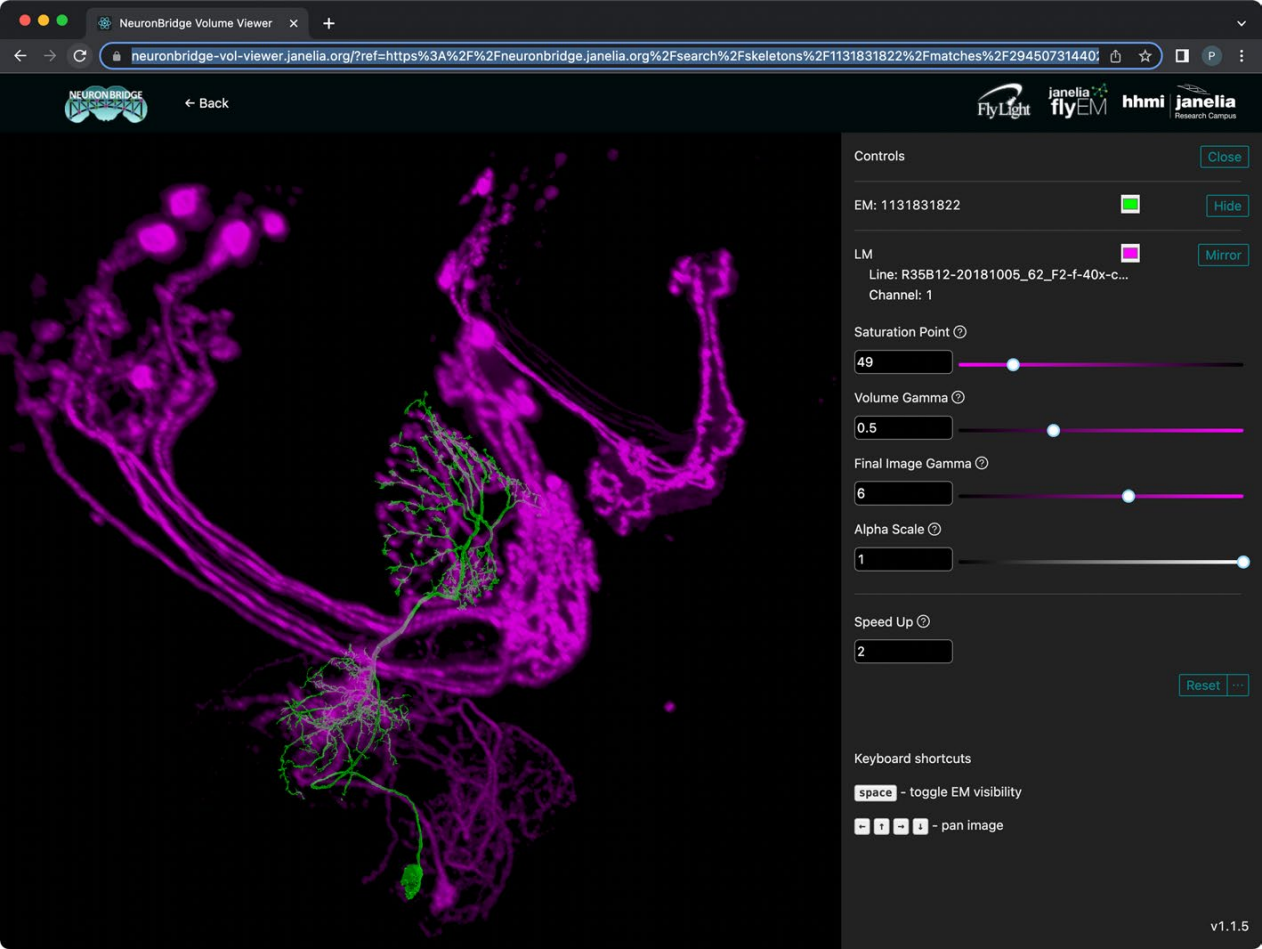
3D visualization of matches in the browser. One channel of the LM image volume is rendered in magenta, and the matching EM neuron skeleton is rendered in green. Controls on the right affect qualities of the rendering like the relative transparency in the LM image volume

NeuronBridge provides two ways a user can manually verify a putative match by viewing the EM neuron skeletons together with LM images from arbitrary viewpoints in 3D. The simplest approach is to click the “View in 3D” button in the detail interface for a search result (Fig. 7). Doing so launches a new browser page that loads the EM neuron skeleton and LM image and renders them in 3D with interactive camera controls (Fig. 10, see also the video in Additional file 1). For LM images we use direct volume rendering, which samples the image volume along rays from the camera, applies a transfer function and lighting model to compute color and opacity at each sample, and composites the results to produce pixels. The EM neuron skeleton is rendered as an opaque surface and blended into the volume rendering with proper occlusion based on its depth map. The renderer uses WebGL2 and achieves interactive performance on modern desktop and laptop computers. The URL for the renderer page is continually updated to encode the details of the current view, so a live rendering can be shared with a collaborator by sharing the URL.

**Fig. 11:**
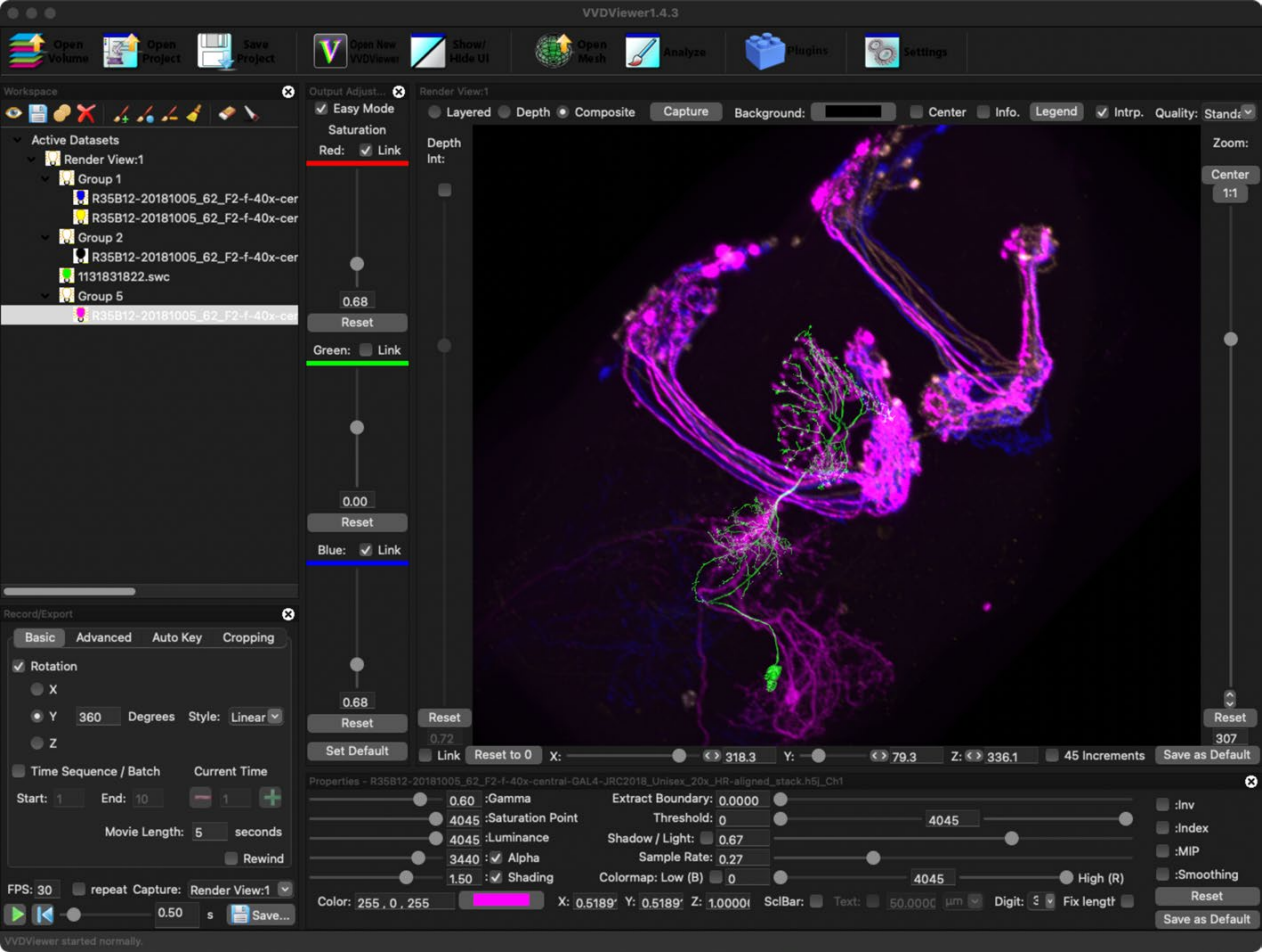
3D visualization of matches in VVDViewer. All three channels of the LM image volume are rendered, in red, green and blue, and the EM neuron skeleton is rendered in aquamarine

A more sophisticated rendering is available by clicking the “Download 3D Files” tab and loading the data into a desktop rendering application like VVDViewer [47] (Fig. 11, see also the video in Additional file 2). VVDViewer renders with slightly higher quality because it uses more bits per voxel of the LM data (12 instead of 8). It also adds several capabilities beyond the browser-based renderer. It can render multiple channels of an LM image simultaneously, better representing the data used by the PPPM matching algorithm. It can render multiple EM skeletons simultaneously, which can be useful for verifying fragmented neurons or for comparing several match results at once. It can also generate animated videos, showing changes to the camera position and other viewing parameters.

### Software architecture

**Fig. 12:**
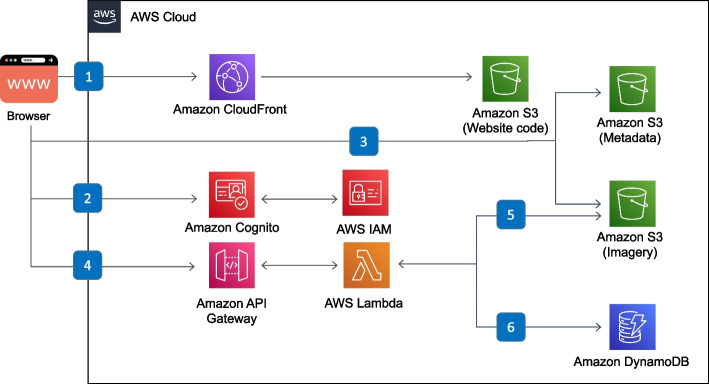
Serverless back-end for precomputed result browsing. The user’s browser communicates with (1) the CloudFront Content Distribution Network (CDN) to retrieve the web client code which is cached from AWS S3. (2) Authentication is done using federated identity providers through Cognito, supporting both email-based accounts and Google accounts, and delegating to IAM for authorization. Precomputed results are (3) statically loaded from public buckets on S3. Dynamic features use (4) an API Gateway endpoint to run a Lambda function, for example to (5) create a ZIP archive of multiple files for download or (6) run prefix searches on DynamoDB

We used fully-managed, serverless AWS services to build the back-end (Fig. 12) of the web application, including Cognito identity federation for authentication, IAM for authorization, Lambda for compute, S3 for storage, and DynamoDB as the database. We used only serverless services to keep operational costs low while allowing us to focus on application logic instead of spending time on server administration.

**Fig. 13:**
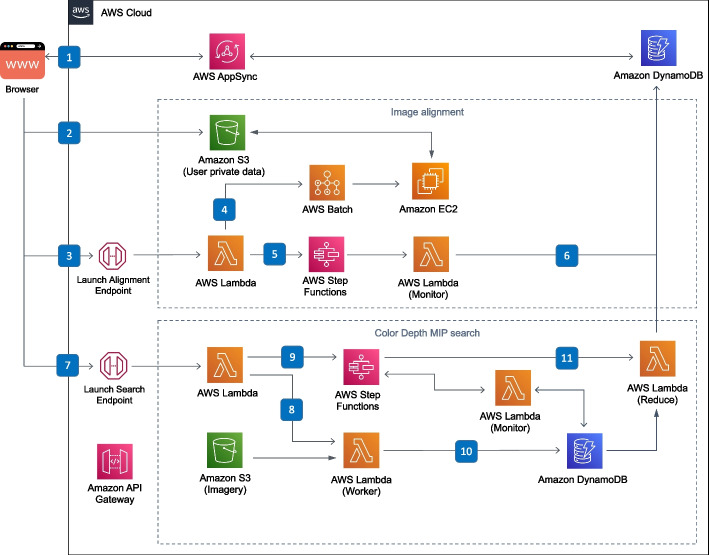
Serverless back-end for custom search. The user initiates a search by uploading an image, and three messages are sent to the back-end: (1) Using AppSync, a new record is created in DynamoDB to track the search workflow, (2) the image is uploaded to a private user folder on S3, and (3) the client starts the alignment process by invoking a Lambda function. (4) A Batch job is created, which allocates an EC2 node and runs the alignment Docker container, fetching the uploaded file from S3, and depositing the aligned CDM images back on S3. (5) A Step Function periodically runs a Lambda to monitor the progress of the Batch job. (6) Once the alignment job is finished, it updates the workflow state in DynamoDB, which notifies the user via AppSync. Back in the browser, the user is asked to create a search mask from one of the resulting CDM images, and this mask is uploaded to S3. (7) The client calls a second endpoint to begin the search, which calls a Lambda that (8) recursively starts the burst-parallel search workers as well as (9) the monitoring Step Function state machine. (10) When a worker finishes, it records its result in a DynamoDB table. Once the monitoring Lambda detects that all workers are finished, (11) it runs the reducer which produces a search result that is updated in the DynamoDB workflow table, notifying the user again through AppSync that the search is complete

Custom search (Fig. 13) consists of two services: image alignment which runs on AWS Batch, and distributed CDM Search which runs on AWS Lambda. The aligner runs on AWS Batch as a Docker container and is monitored by a Step Function which notifies the client when the alignment is completed. To enable efficient searching across large image data sets while keeping costs low, we implemented a burst-parallel [48] search engine built on Lambda and Step Functions [49]. To reduce cold starts when executing searches on AWS Lambda, we also translated the core CDM Search algorithm from Java to JavaScript.

The serverless model is a particularly good fit for the burst-parallel implementation for several reasons. First, massive parallelism is required to quickly search large image libraries, and the ideal level of parallelism is prohibitively expensive in a traditional server-based architecture. The 3000-fold parallelism we achieved with AWS Lambda for a few cents would require one hundred 30-core dedicated servers in a traditional data center deployment. Second, the service has unpredictable usage and can sit idle for extended periods, while at other times has bursts of high activity. This type of usage pattern is directly addressed by a serverless model that can scale to zero. The costs to run this service are low because serverless services bill for compute/storage that is utilized, similar in concept to a traditional HPC compute cluster. In addition, the serverless approach reduced the amount of ongoing maintenance by eliminating server administration. Finally, using serverless Lambda functions allowed us to fully leverage the concurrent throughput of the S3 buckets hosting the images.

The software is reliably deployed through the use of the Serverless Framework, which generates low-level AWS CloudFormation instructions for deployment. Reproducibility and isolation of the deployment is important to being able to run multiple versions of the system at the same time. In a single AWS account, we run a separate instance for each developer, a validation instance for testing before production deployments, a pre-publication instance for internal use, and the production instance that is publicly accessible.

### Data model

**Fig. 14:**
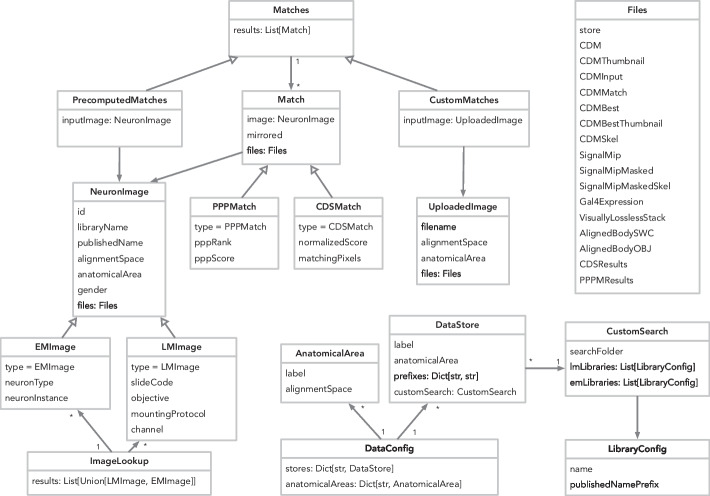
Data model. The DataConfig describes the metadata accessible through the API. Image lookup queries return an ImageLookup object containing neuron images. A NeuronImage has a GUID called id which can be queried for Matches

The data model (Fig. 14) is anchored by a NeuronImage, representing a set of neurons in an alignment space that can be compared with other images in that alignment space, and a set of Matches between NeuronImages, generated by either CDM Search or PPPM. Image metadata is denormalized wherever an image is referenced, to allow clients to fetch a single JSON file instead of making multiple requests. The DataConfig provides summary information, such as the list of possible anatomical areas, as well as providing constants which allow long, common values to be interpolated into JSON values as shorter keys, reducing disk space usage and transfer time. Importantly, this model is trivially extensible with new matching algorithms and new imaging modalities.

All data served by NeuronBridge is available for programmatic access via open data APIs (Open Data API) allowing for reuse and integration. These APIs have already enabled the creation of several third-party applications including the Neuronbridger R API (http://natverse.org/neuronbridger) and FlyBrainLab NeuroNLP [50]. The serverless back-end automatically scales to meet additional load from these API users and imposes rate limits to avoid impacts on overall service performance.

**Fig. 15:**
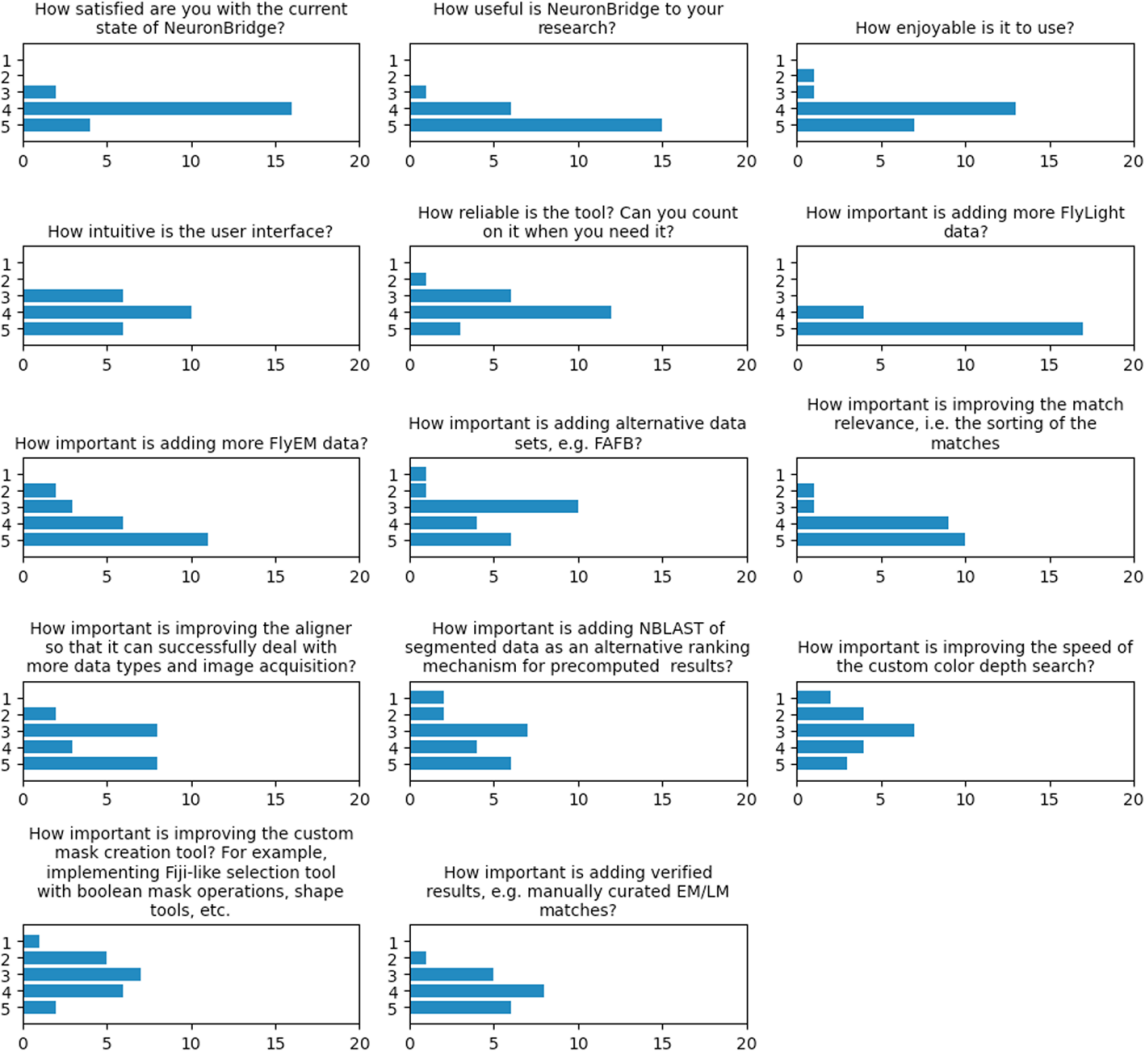
User survey responses. Aggregated responses to the user survey where a value of 1 indicates “Least” and 5 indicates the “Most” response for each question

## Results and discussion

NeuronBridge currently serves over 500 million precomputed matches between 6773 driver lines and 54,061 EM neuron skeletons. In addition, CDM images of these driver lines and neurons have been made available for online custom searching, comprising a data set of 1.2 million CDM images. These searchable 2D images efficiently represent signals extracted from approximately 256 TB of confocal light microscopy and 54 TB of electron microscopy data (Fig. 2) in 241 GB of searchable images, a three orders of magnitude compression which operationalizes the data for neuron searching. We additionally provide aligned EM neuron skeletons in SWC format [51], a standardized format (https://incf.org/swc) for neuron morphologies. We also link to aligned LM images [15] in H5J format (https://data.janelia.org/h5j), so that putative matches can be validated manually in 3D using an external system like VVDViewer [47], to supplement the 3D viewing supported directly in the GUI (3D Visualization).

We intentionally focused NeuronBridge on the use case of finding similar neuron morphologies, so it contains no features allowing users to search or view other types of data such as neuronal circuits, neurotransmitter information, or detailed metadata. The quality of the matches presented by NeuronBridge is bounded by the effectiveness of the underlying matching algorithms. The CDM Search and PPPM algorithms both produce useful and often complementary sets of putative matches [15]. CDM Search struggles with occlusions in the projection dimension, while PPPM has difficulty with segmentation of dense samples, which can cause false positives during search [14]. PPPM results are currently only available for a subset of the Gen1 MCFO data set and exclude the Gen1 Annotator MCFO and Split-GAL4 data sets. NeuronBridge also does not currently load reverse matches (LM$\rightarrow$EM) for PPPM.

The architecture is scalable but limits some of the functionality in ways we anticipated. The choice of using S3 as a match database limits the queries that can be done to lookups by identifier, so users must rely on other websites for metadata-based search. Also, implementing the custom search on top of AWS Lambda limits us to a 15 min execution time per function, though that can be worked around by using a smaller batch size and launching more Lambda functions.

In the future, NeuronBridge could benefit from improved and/or new neuron matching algorithms, additional functionality for match inspection, and any other improvements that address match quality or the core workflows of match browsing, verification, and export. We also expect this resource to grow with additional data over time, including additional EM data sets (e.g. the FANC [5] and FlyWire [7] connectomes), as well as new driver line collections.

We intend for NeuronBridge to remain a very focused tool, and we have therefore intentionally constrained its architecture and implementation to solving the neuron matching use cases. We provide contextual links in cases where other web applications already provide related functionality. For access to source LM images, we link to FlyLight’s anatomy websites (https://flweb.janelia.org, https://gen1mcfo.janelia.org, https://splitgal4.janelia.org). For EM neuron reconstructions we link to neuPrint ([52], https://neuprint.janelia.org). We also provide cross reference links to the Virtual Fly Brain [43] for both EM and LM results. All of these websites link back to NeuronBridge, forming a synergistic ecosystem of tools and data.

All of the software code and data underlying the NeuronBridge service is available for reuse. Up-to-date deployment instructions are maintained on each of our GitHub repositories (Software Repositories) and the entire service could be duplicated elsewhere, for example to serve matches for other data sets. The code is also highly modular so that parts of it can be reused without deploying the entire system. For example, the back-end AWS service implementation could be deployed to integrate CDM Search into another web application. The Apache Spark CDM Search could be reused in a similar manner for an on-premise search solution. The data model and accompanying Python API could be reused for providing programmatic access to neuron morphology matches, including matches from novel matching algorithms that might be developed in the future.

NeuronBridge is an open source project that we developed for the open science community. We are continuing to add new features, most often driven by additional data sets including upcoming FlyLight/FlyEM data sets and connectomes originating outside of Janelia. We also welcome open source community engagement, including code reuse, issue reporting, and code contributions through pull-requests.

We hope that the cloud-based serverless architecture of NeuronBridge, uncommon in open source scientific research software, inspires architectural decisions or code reuse in other tools and platforms for large scientific data analysis. In particular, the burst-parallel image search balances large data analysis with online query capability on interactive timescales, and could be reused to perform many other types of analysis where a large data volume must be fully traversed while a user is waiting.

**Fig. 16:**
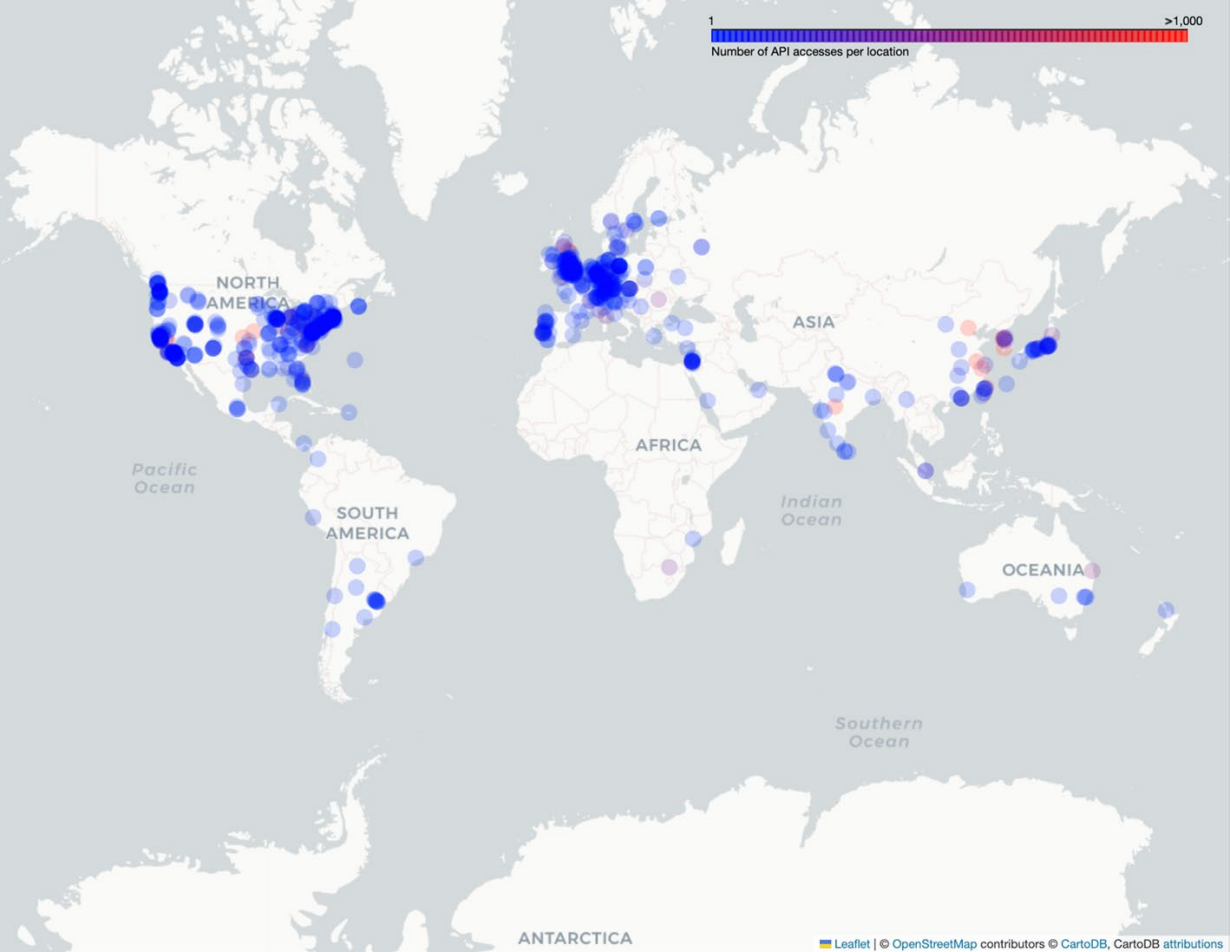
Access map. Depicts access requests to the s3://janelia-neuronbridge-data-prod and s3://janelia-neuronbridge-web-prod buckets during the two year period between 2020-05-08 and 2022-06-08

## Conclusion

The NeuronBridge web application fills a gap created by recently published large data sets including the FlyEM Hemibrain and MANC connectomes and FlyLight image sets for characterizing driver lines. Researchers making use of these connectomes can now efficiently find driver lines that target their neurons of interest, as well as search these data sets to verify cell type identity and driver line expression. The software code underlying the service is modular and can be reused in different ways. In addition, the data powering the web application can be reused either by download or through integration with our Open Data APIs. We believe that these properties will make NeuronBridge an indispensable tool for *Drosophila* neuroscience research.

## Availability and requirements

**Project name** NeuronBridge

**Home page** http://neuronbridge.janelia.org

**Operating system(s)** Platform independent

**Programming language** Java, Javascript, Python

**Other requirements** W3C-compliant web browser

**License** BSD-3-Clause license

**Any restrictions to use by non-academics** None.

## Additional material

### Open data API

The S3 bucket containing the NeuronBridge matches forms an open REST API. Key structures are arranged such that they can be queried predictably. We use the standard JSON format and publish a schema for each document type. The schemas are versioned so that future changes are not breaking. All of the endpoints below are relative to the bucket root at s3://janelia-neuronbridge-data-prod

- current.txt
  Returns the current version, to be used in subsequent API calls as <VER>. Older versions of the metadata are preserved so a client can choose to request older data for compatibility or other reasons.
- <VER>/schemas/
  A prefix containing the version-specific JSON schemas for all objects in the data model.
- <VER>/config.json
  Returns a configuration object containing base URL prefixes and other metadata necessary for programmatic use of the following API. Follows the JSON schema defined by DataConfig.json.
- <VER>/DATA_NOTES.md
  Returns a Markdown document containing the data release notes.
- <VER>/metadata/by_body/<body_id>.json
  For a given body ID (i.e., the identifier for a specific EM neuron reconstruction), returns metadata such as a path to a representative image, neuron names, etc. Follows the JSON schema defined by ImageLookup.json.
- <VER>/metadata/by_line/<line_id>.json
  For a given driver line identifier, returns metadata including a list of images from the line (most lines have been characterized multiple times), representative images, and other attributes. Follows the JSON schema defined by ImageLookup.json.
- <VER>/metadata/cdsresults/<image_id>.json
  For a CDM neuron image provided by either the by_body or by_line endpoints, returns a list of matching images as computed by CDM Search. Neuron images have Globally Unique Identifiers (GUID) which persist across data versions, making it easier to link to data and results. Follows the JSON schema defined by PrecomputedMatches.json.
- <VER>/metadata/pppmresults/<image_id>.json
  For a CDM neuron image provided by either the by_body or by_line endpoints, returns a list of matching images as computed by PPPM search. Follows the JSON schema defined by PrecomputedMatches.json.

### User survey results

We conducted a survey to assess users’ satisfaction with the service and determine which features and improvements should be prioritized. We received 22 responses to the survey during the 2021 calendar year (Fig. 15). By far, additional data was the most important aspect for users, which led us to prioritize the addition of matches for the FlyEM MANC and additional FlyLight data sets as they were released.

### Usage statistics

As of January 2024, there are over 1600 registered users of NeuronBridge. During the calendar year of 2023, the site received 75,256 page views, users uploaded 880 images, and ran 933 custom searches against the uploaded data. The S3 API received 328.4 million requests from 477 distinct organizations, excluding our own. Since its initial deployment, NeuronBridge has been accessed from 72 countries around the world (Fig. 16).

### Data repositories

The data is available in the following public S3 buckets:

- s3://janelia-neuronbridge-data-prod CDM Search and PPM match metadata in standard JSON format. The bucket structure is fully described in the Open Data API section. The JSON metadata refers to images in the buckets below.
- s3://janelia-flylight-color-depth
  CDM images in PNG format for display are found within each <AlignmentSpace>/<Library> folder. This bucket also contains the same images in TIFF format for searching under <AlignmentSpace>/<Library>/searchable_neurons. The TIFF files are grouped into prefix groups for better scalability when searching with thousands of AWS Lambda functions.
- s3://janelia-ppp-match-prod
  PPPM result images in PNG format. Imported from the published PPPM results [14].

### Software repositories

- Web client implementation
  - Source code repository: https://github.com/JaneliaSciComp/neuronbridge
  - Archived code at time of publication: https://doi.org/10.5281/zenodo.10541060
- Back-end implementation
  - Source code repository: https://github.com/JaneliaSciComp/neuronbridge-services
  - Archived code at time of publication: https://doi.org/10.5281/zenodo.10541063
- Color Depth MIP search library and Apache Spark implementation
  - Source code repository: https://github.com/JaneliaSciComp/colormipsearch
  - Archived code at time of publication: https://doi.org/10.5281/zenodo.10541073
- Scripts related to precomputed CDM results
  - Source code repository: https://github.com/JaneliaSciComp/neuronbridge-precompute
  - Archived code at time of publication: https://doi.org/10.5281/zenodo.10541087
- Aligner implementation
  - Source code repository: https://github.com/JaneliaSciComp/neuronbridge-aligners
  - Archived code at time of publication: https://doi.org/10.5281/zenodo.10541088
- Python API
  - Source code repository: https://github.com/JaneliaSciComp/neuronbridge-python
  - Archived code at time of publication: https://doi.org/10.5281/zenodo.10541244
- 3D visualization
  - Source code repositories: https://github.com/JaneliaSciComp/neuronbridge-vol-viewerhttps://github.com/JaneliaSciComp/web-vol-viewerhttps://github.com/JaneliaSciComp/web-h5j-loader
  - Archived code at time of publication: https://doi.org/10.5281/zenodo.10541250https://doi.org/10.5281/zenodo.10530403https://doi.org/10.5281/zenodo.10541255

### Supplementary Information

**Additional file 1**. Screen capture movie showing a user clicking on the “View in 3D” button to inspect a putative match between a EM neuron skeleton and LM image in 3D.

**Additional file 2**. Screen capture movie showing a user inspecting a putative match in VVDViewer after downloading the EM neuron skeleton and LM image using the “Download 3D Files” tab.

## Data Availability

All public data in NeuronBridge (i.e., excluding data uploaded by users) is available for access on AWS S3. In addition to acting as a database back-end for the NeuronBridge browser application, the data on S3 serves as an Open Data API (Open Data API). All of the data is licensed under the Creative Commons Attribution 4.0 International (CC BY 4.0) license.

## Abbreviations

API: Application Programming Interface
AWS: Amazon Web Services
CDM: Color Depth MIP
CDN: Content Distribution Network
CMTK: Computational Morphometry Toolkit
DSLT: Direction Selective Local Thresholding
EM: Electron microscopy
FIB-SEM: Focused Ion Beam Scanning Electron Microscope
GB: Gigabyte
GPU: Graphics Processing Unit
GUI: Graphical User Interface
H5J: File format for light microscopy images
HPC: High Performance Computing
HTTP: Hyper-Text Transfer Protocol
JSON: JavaScript Object Notation
LM: Light microscopy
MANC: FlyEM Male Adult Nerve Cord
MIP: Max intensity projection
MCFO: Multi-Color Flip Out
PPPM: PatchPerPix Match
REST: Representational State Transfer
SWC: File format for reconstructed neuron morphologies
TB: Terabyte
UAS: Upstream Activating Sequence
VNC: Ventral nerve cord of *Drosophila*
ZIP: Compressed file format
